# Need for international consensus on diagnostic criteria for fetal alcohol spectrum disorders (FASD): Commentary on Myers et al., “Comparing rates of agreement between different diagnostic criteria for fetal alcohol spectrum disorder: A systematic review”

**DOI:** 10.1111/acer.70095

**Published:** 2025-06-07

**Authors:** Elizabeth Jane Elliott

**Affiliations:** ^1^ Paediatrics and Child Health, Faculty of Medicine and Health University of Sydney Sydney Australia; ^2^ Sydney Children's Hospitals Network Sydney Australia; ^3^ NSW Fetal Alcohol Spectrum Disorder Assessment Service Sydney Australia; ^4^ National Health and Medical Research Council of Australia Sydney Australia

As highlighted in the systematic review by Myers et al. (2025), multiple different sets of criteria have spawned internationally for the diagnosis and classification of fetal alcohol spectrum disorder (FASD), with variable agreement between criteria. A lack of international agreement on diagnostic criteria has significantly impeded global understanding of the disorder by clinicians and limited meaningful collaborative research. In short, if we all use different diagnostic methods and terminology, we risk comparing apples with oranges.

Alcohol embryopathy was first described as fetal alcohol syndrome—or FAS—in the English literature by Jones and Smith (1973). They described children whose mothers used excessive alcohol in pregnancy and who exhibited characteristic facial features, growth restriction, congenital anomalies, and developmental delay. Later recognition that the phenotype associated with prenatal alcohol exposure (PAE) was very heterogeneous—depending not only on the pattern of PAE, but maternal and fetal genetics, maternal health and age, and a host of other complex factors (May & Gossage, 2011), led to the use of terms such as fetal alcohol effects (FAE) and ultimately FASD. Inherent in the use of the term FASD is acknowledgment that PAE has a ‘spectrum’ of effects; for example, first‐trimester PAE may cause facial dysmorphology and congenital anomalies associated with functional neurodevelopmental problems, while exposure at any time in pregnancy may result in neurodevelopmental problems in the absence of physical features.

Diagnostic guidelines act as a road map to lead clinicians along a pathway to assessment and diagnosis of FASD, but even within countries different approaches are used: the 4‐digit code (Astley Hemmingway, 2024), Institute of Medicine criteria (Hoyme et al., 2016) and CDC criteria (CDC, 2024) are all used in the United States. As outlined by Myers et al. (2025) guidelines have also been published in Canada (Cook et al., 2016), Australia (Bower & Elliott, 2016), New Zealand (Aotearoa (NZ) FASD Guidelines Development Project Team, 2024), Scotland (Scottish Intercollegiate Guidelines Network, 2019), and Germany (Landgraf et al., 2024). It is likely these were developed to overcome the perceived complexity of some criteria for use in clinical settings, and the perceived lack of rigor in others, including absence of cut‐points for clinical significance in growth or neurodevelopmental function. The Australian and UK guidelines were derived from the Canadian criteria, with the benefit of allowing a consistent approach internationally.

Terminology is also a barrier to international consistency. An alphabet of acronyms is used to categorize outcomes following PAE, including FASD, FAS, partial FAS, alcohol‐related neurodevelopmental disorder (ARND), alcohol‐related birth defects (ARBD), and static encephalopathy. To add to the fray, several guidelines use FASD (FASD with three sentinel facial features or FASD with fewer than three sentinel features) as a diagnostic term (Aotearoa (NZ) FASD Guidelines Development Project Team, 2024; Bower and Elliott, 2016; Cook et al., 2016; Scottish Intercollegiate Guidelines Network, 2019). Conversely, the CDC states that “the term FASD is not meant for use as a clinical diagnosis” and “should only be used as an umbrella term within which different sub‐groups of FASD are identified.” The 4‐digit code takes a different approach to classification, allocating a severity score from 1 to 4 for each of growth deficiency, facial phenotype of FASD, structural and functional brain abnormalities and prenatal alcohol exposure (Astley Hemmingway, 2024). This results in over 200 possible code combinations and numerous diagnostic categories, five of which fall under the broad umbrella of FASD.

The key challenge in diagnosing FASD is the absence of a definitive diagnostic test—including any accurate biological marker (Popova et al., 2023). This also applies to other neurodevelopmental disorders such as attention‐deficit hyperactivity disorder (Wolraich et al., 2019), autism spectrum disorder (Goodall et al., 2023), and sleep disorder (Ogundele & Yemula, 2022), in which diagnosis is made after clinical assessment—using a check list of criteria or an algorithm to document the typical “symptom complex.” In considering any of these disorders, clinicians must exclude differential diagnoses, including genetic disorders, and document potential prenatal and postnatal contributors to neurodevelopmental impairment such as complex early life trauma, brain injury, or metabolic disorders (Bower and Elliott, 2016). They must also be cognisant of different clinical presentations at different ages, the overlap between clinical phenotypes in neurodevelopmental disorders, and possible coexisting diagnoses.

Unlike many neurodevelopmental disorders, for which etiology is unknown, FASD is attributed to PAE, a well‐established teratogen and neurotoxin whose effects can be reliably reproduced in preclinical models (Popova et al., 2023). Confirming PAE is often difficult because many children with FASD grow up in out‐of‐home care. When obstetric records lack reliable information on PAE, review of child protection, justice, and other records, or obtaining firsthand witness reports is required.

Although not explicitly stated by Myers et al. (2025), most diagnostic criteria for FASD are more similar than different; all include the criteria of PAE and severe neurodevelopmental impairment, and all except DSM‐5 include facial dysmorphology. All suggest documenting—but do not necessarily include as essential diagnostic features—growth and congenital anomalies. However, the cut‐offs used for determining functional impairment, the domains of function recommended for assessment, and the inclusion of criteria such as growth differ.

Few guidelines consider the challenges of assessing infants or adults or the changes in the behavioral and physical phenotype that may occur over the lifespan. Few offer approaches that truly consider the cultural context of people being assessed for FASD, particularly First Nations populations (Aotearoa (NZ) FASD Guidelines Development Project Team, 2024). This is important for several reasons. First, the prevalent assumption that “FASD is a First Nations problem” may lead to stigmatization and racism and must be discounted. Second, there is a lack of standardized, validated, and language‐independent tools for assessing neurodevelopment for many First Nations peoples and a lack of normative data, including for facial features. Third, the approach to the diagnosis and management of FASD must be codesigned with Indigenous people, culturally safe, and adhere to traditional protocols. To address these issues an Aboriginal author has published the Australian Indigenous FASD Framework, which suggests an approach to FASD that blends Aboriginal and Torres Strait Islander and Western wisdom, takes a respectful healing‐informed approach, and facilitates equitable access to strength‐based assessment, diagnosis, and support that engenders hope. (Hewlett et al., 2023).

For most neurodevelopmental diagnoses, clinicians globally use diagnostic criteria included in t*he Diagnostic and Statistical Manual of Mental Disorders (DSM‐5‐TR) (*American Psychiatric Association, 2024) or the International Classification of Diseases for Mortality and Morbidity Statistics (ICD‐11) (WHO, 2024), which ensures a consistent approach. However, the name FASD is not listed in the DSM‐5‐TR, which instead includes Neurobehavioral Disorder Associated with Prenatal Alcohol Exposure (ND‐PAE) as a psychiatric diagnosis that requires evidence of PAE and impairment in the three functional domains of cognition, self‐regulation, and adaptive functioning (Doyle & Mattson, 2015). Some, not all, people with a diagnosis of FASD will fulfill criteria for ND‐PAE. Similarly, FASD is not included in the ICD‐11, which lists only FAS (code LD2F.00) as both a ‘malformation syndrome caused by maternal consumption of alcohol during pregnancy’ and a ‘neurodevelopmental syndrome due to prenatal alcohol exposure’ (code 6AOY). Both these classification systems differ from those used in existing diagnostic guidelines and provide yet more alternatives for consideration by clinicians.

Standardizing the diagnostic approach is important to guide clinical care and enable rigorous scientific research. Considering this, Myers et al. asked an important question about the agreement between different diagnostic criteria for FASD. They conducted the first systematic review of peer‐reviewed publications that directly compare the likelihood of receiving a diagnosis of FASD using one set of criteria versus another (eight studies reporting 17 comparisons), published in *Alcohol: Clinical and Experimental Research* (Myers et al., 2025). In a move away from past studies, which have simply applied different diagnostic criteria in the same population, the authors extracted data to allow calculation of the percentage agreement and disagreement for a FASD diagnosis using two different sets of criteria and used meta‐analyses to derive a composite estimate of agreement when making multiple comparisons between studies.

The percentage agreement between eight sets of criteria for a diagnosis of FASD (or no diagnosis) ranged from 53.7% to 91% (Myers et al., 2025), not surprising considering both the disparity and similarity between international guidelines. For example, the percentage agreement for a FASD diagnosis between Australian and Canadian criteria, on which Australian criteria are based, was 85.2%. On the other hand, there was considerable variation in the percentage agreement for diagnosis (59.4%–82.5%) when the Canadian criteria were compared with five other sets of criteria. In another example, a child diagnosed using the IOM criteria had a one in four chance of receiving a different diagnosis when four other sets of criteria were used.

So, what are the implications of these findings? The lack of agreement between widely used diagnostic systems is consequential. Without an accurate diagnosis, clinicians cannot provide individuals and families with an understanding of the origin of their challenges. Failure to diagnose may lead to missed opportunities for support to assist a mother to abstain from alcohol in a future pregnancy. It may restrict families from accessing appropriate services, supports, and optimal treatment, or from applying for remedial education or government support. It may jeopardize judicial outcomes. Confusion among clinicians about how to make the diagnosis will inevitably result in diagnostic delays, misdiagnosis, or underdiagnosis (Chasnoff et al., 2015) with significant impacts on families (Phillips et al., 2022). Importantly, a diagnosis—and the earlier the better—provides a window of opportunity for early intervention to address the severe and pervasive neurodevelopmental problems that are characteristic of FASD.

Assessments conducted during the diagnostic process also allow us to identify the individual strengths and qualities of people living with FASD and to offer the necessary supports to help them build on these. In an era of limited healthcare resources, the diagnosis of FASD is costly and time‐consuming, requiring multidisciplinary input from a range of skilled and FASD‐informed clinicians. As Myers et al. (2025) states, the “demand for FASD diagnostic services far exceeds current capacity in many countries,” so surely we should use our precious resources wisely. A broad choice of diagnostic criteria may leave clinicians confused as to what diagnostic approach is most accurate and clinically useful. Furthermore, in the context of research, a lack of agreement between diagnostic criteria makes it impossible to make global comparisons on prevalence rates for FASD, the frequency of neurodevelopmental impairments, and the need for and response to therapies, supports and strategies for prevention. To this end the National Institute on Alcohol Abuse and Alcoholism (NIAAA) has convened a Consensus Committee with a remit to establish an international classification system for FASD to improve the comparability of research internationally (Mooney et al., 2022). The development of clinical guidelines is beyond NIAAA's mission and is not part of these efforts. Significant additional work would be required to achieve a consistent clinical approach to diagnosis that is agreed for use internationally and applicable in different contexts.

We cannot afford to keep comparing apples with oranges. We need international consensus on a “Gold Standard” diagnostic approach to FASD. The future challenge for the research community will be to prospectively obtain the evidence needed to enable us to better understand the way in which PAE impacts the fetal brain at different gestational ages and to refine the classification of FASD accordingly. We must also consider the impact of genetic variants, simultaneous exposure to alcohol and other drugs, complex early life trauma, and variable epigenetic responses to PAE on the FASD phenotype. Considering the inadequacy of clinical services internationally to deal with demand, clinical capacity must be built for diagnosing and managing FASD, especially in primary care and rural settings, and new technologies including telehealth embraced. In addition, we must explore new ways to diagnose FASD earlier, because experience in other neurodevelopmental disorders confirms that early intervention can change life trajectories. Novel approaches to diagnosis might include use of web‐based tools (Hyland et al., 2023) and exploring the utility of biological markers, including epigenetic signatures, as screening tools for PAE (Popova et al., 2023). For example, in a proof of concept study, markers of PAE have been identified in baby teeth (Montag et al., 2022) Sophisticated 3‐D imaging may help delineate facial characteristics associated with FASD or PAE—apart from the three sentinel features—and thus help identify infants at most risk of FASD (Muggli et al., 2017, 2025). Machine learning may also facilitate assessment and diagnosis (Suttie et al., 2024); however, the limited resources available both for clinical services and research will restrict the rate at which these innovations can be explored.

## CONFLICT OF INTEREST STATEMENT

The author has no conflict of interests.
